# Ruxolitinib Adherence in Myelofibrosis and Polycythemia Vera: the “RAMP” Italian multicenter prospective study

**DOI:** 10.1007/s00277-024-05704-0

**Published:** 2024-03-13

**Authors:** F. Palandri, G. Auteri, E. Abruzzese, G. Caocci, M. Bonifacio, F. Mendicino, R. Latagliata, A. Iurlo, F. Branzanti, B. Garibaldi, M. M. Trawinska, D. Cattaneo, M. Krampera, O. Mulas, E. A. Martino, M. Cavo, N. Vianelli, S. Impera, F. Efficace, F. Heidel, M. Breccia, E. M. Elli, G. A. Palumbo

**Affiliations:** 1 IRCCS Azienda Ospedaliero-Universitaria Di Bologna, Istituto Di Ematologia “Seràgnoli”, Bologna, Italy; 2 Dipartimento Di Medicina Specialistica, Diagnostica E Sperimentale, Università Di Bologna, Bologna, Italy; 3 Hematology, S.Eugenio Hospital, Tor Vergata University, ASL Roma2, Rome, Italy; 4 Hematology Unit, Department of Medical Sciences, University of Cagliari, Cagliari, Italy; 5 Hematology and Bone Marrow Transplant Unit, Section of Biomedicine of Innovation, Department of Engineering for Innovative Medicine, University of Verona, Verona, Italy; 6 U.O.C. Di Ematologia, Department of Hemato-Oncology, Azienda Ospedaliera Annunziata, Cosenza, Italy; 7 Hematology Unit, Ospedale Belcolle, Viterbo, Italy; 8 Hematology Division, Foundation IRCCS Ca’ Granda Ospedale Maggiore Policlinico, Milan, Italy; 9 Postgraduate School of Hematology, University of Catania, Catania, Italy; 10 Department of Hematology, ARNAS Garibaldi, Catania, Italy; 11 Data Center and Health Outcomes Research Unit, Italian Group for Adult Hematologic Diseases (GIMEMA), Rome, Italy; 12 Hematology, Hemostasis, Oncology and Stem Cell Transplantation, Hannover Medical School (MHH), Hannover, Germany; 13 Division of Cellular Biotechnologies and Hematology, University Sapienza, Rome, Italy; 14 Divisione di Ematologia e Unità Trapianto di Midollo, Fondazione IRCCS San Gerardo Dei Tintori, Monza, Italy; 15 Dipartimento di Scienze Mediche, Chirurgiche e Tecnologie Avanzate “G.F. Ingrassia”, Università Di Catania, Catania, Italy

**Keywords:** Adherence to medication, Ruxolitinib, Myelofibrosis, Polycythemia Vera, Distress, Adherence

## Abstract

Ruxolitinib is beneficial in patients with myelofibrosis (MF) and polycythemia vera (PV). Information on ruxolitinib adherence is scant. The Ruxolitinib Adherence in Myelofibrosis and Polycythemia Vera (RAMP) prospective multicenter study (NCT06078319) included 189 ruxolitinib-treated patients. Patients completed the Adherence to Refills and Medications Scale (ARMS) and Distress Thermometer and Problem List (DTPL) at the earliest convenience, after registration in the study, and at later timepoints. At week-0, low adherence (ARMS > 14) and high distress (DT ≥ 4) were declared by 49.7% and 40.2% of patients, respectively. The main reason for low adherence was difficult ruxolitinib supply (49%), intentional (4.3%) and unintentional (46.7%) non-take. In multivariable regression analysis, low adherence was associated to male sex (*p* = 0.001), high distress (*p* < 0.001), and treatment duration ≥ 1 year (*p* = 0.03). Over time, rates of low adherence and high distress remained stable, but unintentional non-take decreased from 47.9% to 26.0% at week-48. MF patients with stable high adherence/low distress were more likely to obtain/maintain the spleen response at week-24. Low adherence to ruxolitinib represents an unmet clinical need that require a multifaceted approach, based on reason behind it (patients characteristics and treatment duration). Its recognition may help distinguishing patients who are truly refractory and those in need of therapy optimization.

**Supplementary Information:**

The online version contains supplementary material available at 10.1007/s00277-024-05704-0.

## Introduction

Myelofibrosis (MF) is a Philadelphia-negative myeloproliferative neoplasm (MPN) characterized by splenomegaly, systemic symptoms, blood cell abnormalities and a tendency to develop thrombotic/hemorrhagic complications and evolution into Acute Myeloid Leukemia (AML), resulting in significantly reduced survival expectation [1–3].

Polycythemia Vera (PV) is a classical MPN characterized by an abnormally elevated red blood cell production caused by the acquisition of somatic mutations in the JAK2 gene, which drives the normal erythropoiesis and myelopoiesis. Like MF, PV is burdened by important systemic symptoms and reduced quality of life. Also, PV has an increased risk of thrombosis and evolution into post-PV MF and/or AML [4–7].

Ruxolitinib is a *JAK1/2* inhibitor indicated in MF patients with splenomegaly and/or symptoms and in PV patients who are resistant or intolerant to hydroxyurea. The registrative COMFORT studies proved clear superiority in spleen and symptoms responses over control arms for MF patients [8–10]. RESPONSE studies showed the efficacy of ruxolitinib in controlling hematocrit, leukocyte and platelet count, in PV patients with or without splenomegaly, after hydroxyurea failure [11, 12].

The efficacy of ruxolitinib is based on continuous administration, as a ruxolitinib discontinuation syndrome, characterized by rapid re-expansion of splenomegaly and symptoms, is observed in MF when the drug is stopped [13]. Additionally, the discontinuation of ruxolitinib in PV usually lead to increase of hematocrit values and re-occurrence of systemic symptoms [14].

It is known that there is a dose–response effect in ruxolitinib-treated patients, with doses lower than 10 mg twice daily being associated with lower responses in MF [15].

Poor adherence may increase treatment failure [16] ad it was recently observed that around one third of the MF patients report an inadequate adherence to ruxolitinib [17]. However, there is dearth of evidence-based data on the role adherence to ruxolitinib therapy in the wider MPN patient population.

The “Ruxolitinib Adherence in Myelofibrosis and Polycythemia Vera” (RAMP) multicenter prospective study was designed to evaluate the incidence of low adherence to ruxolitinib therapy, and the factors associated with it, including the psychological distress. Also, the modification over time of self-reported adherence and distress were monitored and correlated with spleen responses in MF patients.

## Materials and methods

### Study design

The RAMP study (NCT06078319) included MF and PV patients diagnosed between January 1985 and December 2021 in 9 academic Hematology Centers (Appendix). Centers collectively submitted the requested clinical, laboratory information and results of self-reported questionnaires. The total number of medical files was reported by each center by data input into an electronic database after de-identification of the patients with an alphanumeric code to protect personal privacy.

Data collected included patient demographics, instruction, medications, clinical/laboratory tests at diagnosis and during follow-up, type of therapies, death and causes of death. Any treatment decision was at the physician’s discretion independently from participation to this study. After the first data entry, the follow-up information was revised.

Patients completed the validated Adherence to Refills and Medications Scale (ARMS) [18] and Distress Thermometer and Problem List (DTPL) [19] at the earliest convenient time after registration in this study (i.e., week-0), irrespective of the date of ruxolitinib start. In no patient week-0 corresponded to the first ruxolitinib intake. ARMS and DTPL evaluations were repeated at week 4, 8, 12, 24 and 48.

The ARMS consists of 12 items rated on a four-point Likert scale ranging from 1 (never) to 4 (always). Lower scores indicate better adherence. Eight questions investigate more specifically intentional (Q2, Q5, Q6, Q7) or non-intentional (Q1, Q4, Q8 and Q10) non-adherence, while four questions investigated logistical (Q3, Q9, Q12) or financial (Q11) aspects of drug supply.

The ARMS questionnaire has already been used in previous studies of patients with hematologic malignancies receiving chronic therapies, showing the value of this measure in capturing adherence to therapy in these settings [20].

Patients were asked to describe the magnitude of emotional distress they had experienced in the last week by indicating, on a visual analogue scale (a drawn thermometer), a number ranging from 0 (no emotional distress/stress) to 10 (maximum emotional distress/stress). From 4 to 6 a moderate level of distress is detected, finally from 7 to 10 the distress is high [21]. Patients are also asked to indicate which of the problems, presented in a problem list and grouped in 5 categories (practical, relational, emotional, spiritual, physical-functional), have been predominant [19, 22].

### Definitions

MF and PV were diagnosed according to the 2016 WHO classification [23]. MF risk category was assessed at week-0 according to the DIPSS [24]. Spleen response was evaluated in patients with MF as defined by the IWG-MRT criteria [25]. Patient-reported symptoms were evaluated by the validated MPN10-Total Symptoms Score (TSS) [26].

Different ARMS cut-off values have been used in different cohorts to categorize patients according to low and high adherence [27–29]. For the purpose if this study, the cut-off value of 14 was selected as it was the mean ARMS score in our cohort. The DT cut-off value of 4 was chosen to distinguish patients with high or low distress, according to standard definition [21].

### Ethical aspects

The RAMP was an academic study performed in accordance with the guidelines of the IRBs of the participating centers and the standards of the Helsinki Declaration. All patients provided written informed consent. The promoter of this study was the IRCCS Azienda Ospedaliero-Universitaria S. Orsola-Malpighi, Bologna, which obtained the approval by the Area Vasta Emilia Centro (AVEC) Ethics Committee (Approval file number: 1064/2020/Oss/AOUBo). The study was also approved by the local Ethics Committee of participating Centers.

### Statistical analyses

Statistical analysis was performed at the biostatistics laboratory of the MPN-Unit, IRCCS Azienda Ospedaliero-Universitaria S. Orsola-Malpighi, Bologna.

Continuous variables were summarized by their median and range, while categorical variables by count and frequency (%) of each category. ARMS and DT scores were summarized by their mean and standard deviation (SD). Association between categorical variables was tested by the χ2 test.

To assess factors associated with low adherence and high distress, the following week-0 variables selected on the basis of clinical plausibility, were explored using a logistic regression model: sex, age > 70 years, MF diagnosis (vs PV), intermediate-2/high DIPSS risk (vs intermediate-1/low risk), TSS ≥ 20, palpable spleen, presence of caregiver, professionally active, low educational level, high distress, low adherence, need for concomitant therapies, intake of ≥ 6 tablets/day excluding ruxolitinib, > 1 year from start of ruxolitinib to week-0.

Regressors associated respectively with low adherence and distress with *p* < 0.05 in univariate analysis (UVA), were jointly tested in a multivariable analysis (MVA) in a linear logistic regression model. In addition, by univariate Cox proportional hazards models, we evaluated associations between death/discontinuation and week-0 low adherence and high distress.

Variations in spleen response rates between week-0 and week-24 in patients with MF were assessed using the McNemar test.

For all tested hypotheses, two-tailed p-values < 0.05 were considered significant. All statistical analyses were performed using STATA Software, 15.1 (StataCorp LP, College Station TX, USA).

## Results

### Study population

Between June 2020 and May 2022, 189 patients completed at least one ARMS and DTPL test. Out of 189, 141 patients had a MF, and 48 had a PV. Patients’ characteristics are summarized in Table 1. Interestingly, median TSS was comparable across the two diseases with around 25% of MF and PV patients having a TSS ≥ 20.

**Table 1:** Characteristic at first administration of the ARMS/DTPL questionnaires

Characteristic at week-0	PV (n. 48, 25.4%)	MF (n. 141, 74.6%)	*p*-value
Male sex, no. (%)	31 (64.6%)	81 (57.5%)	0.39
Age (years), median (range)	65.7 (37–84)	71.1 (33.7–88.9)	**0.008**
> 65 years, no. (%)	26 (54.2%)	102 (72.3%)	**0.02**
> 70 years, no. (%)	17 (35.4%)	78 (55.3)	**0.02**
Median time on ruxolitinib, years (range)	2.57 (0.5–13.4)	2.4 (0.05–13.1)	0.9
Median time from diagnosis to w0, years (range)	9.8 (0.3–28.9)	3.6 (0.1–26.7)	** < 0.001**
> 1 year from ruxolitinib start, no. (%)	40 (83.3%)	110 (78.0%)	0.43
Palpable Spleen			
median cm below left costal margin (BLCM), median (range)	0 (0–8)	3 (0–30)	** < 0.001**
Spleen ≥ 10 cm BLCM, no. (%)	0	24 (17.0%)	**0.002**
BMI, median (range)	26 (19.5–37)	24 (16–35.5)	**0.002**
Hemoglobin, median (range), g/dL	13.8 (9.2–16.2)	11.6 (6.4–17.6)	** < 0.001**
Platelet count, median (range), × 10^9^/L	381 (122–1539)	314 (42–1425)	0.06
Leukocyte count, median (range), × 10^9^/L	9.3 (3.7–29.2)	10.55 (3.1–92.5)	0.1
TSS, median (range)	27 (0–70)	26 (0–100)	0.84
TSS ≥ 20, no. (%* on 152 available)*	10 (25%)	31 (28.4%)	0.68
Peripheral blasts, median (range), %	0	0 (0–10)	**0.007**
Spleen response, no. (% on 141 MF evaluable patients)	n.a	65 (46.1)	
Main reason for ruxolitinib start in PV patients, no. (%)			
Intolerance to hydroxyurea	22 (45.8%)	n.a	
Resistance to hydroxyurea	26 (54.2%)	n.a	

*PV* polycythemia vera, *MF* myelofibrosis, *BMI* body mass index, *TSS* total symptom score, *n.a.* not applicable

The first questionnaire was completed within 1 year from ruxolitinib start in 127 patients (67.2%), and therefore evaluated early adherence to ruxolitinib. In the 62 (32.8%) patients who entered the study after more than one year of ruxolitinib therapy, late adherence was evaluated. During the 48-week observation time, 8 patients discontinued ruxolitinib and 10 died. Thirty-two (16.9%) patients refused to fill the questionnaires and dropped out of the study at various times. Patient disposition is summarized in Supplemental Fig. 1.

Overall, 138 (73%) patients, defined “full-completers”, completed all tests from week-0 to week-48. The percentage of “full completers” was slightly higher in PV (38/48, 79.2%) than in MF (100/141, 70.9%).

### Basic patients’ information

At week-0, a preliminary questionnaire was administered to all patients to collect key social information. Overall, 46.5% of the patients declared to have a low level of education (middle school or lower) (Supplemental Table 1).

Notably, 80.4% of patients received other drugs besides ruxolitinib, and 94 (49.7%) were taking more than 6 tablets per day (ruxolitinib excluded). However, almost all recognized the importance of correct ruxolitinib intake for improving their health status. Finally, being followed at all times by a fixed team of hematologists was found to be critical in generating a satisfactory patient-doctor relationship.

### Ruxolitinib adherence at week-0

At week-0, the mean ARMS was 14.35 (SD, 2.02). Overall, 94 (49.7%) patients declared a low adherence (ARMS score > 14).

Table 2 shows the main differences in patients’ responses to the 12-ARMS items, according to low or high adherence to ruxolitinib.

**Table 2:** Responses to ARMS items according to high or low adherence at week 0

Questions	LOW-ADH (94)		HIGH-ADH (95)	
	Score = 1	Score ≥ 2	Score = 1	Score ≥ 2
1. How often do you forget to take your medicine?	58 (61.7%)	36 (38.3%)	89 (93.7%)	6 (6.3%)
2. How often do you decide not to take your medicine?	88 (93.6%)	6 (6.4%)	94 (98.9%)	1 (1.1%)
3. How often do you forget to get prescriptions for your medicine?	91 (96.8%)	3 (3.2%)	95 (100%)	0
4. How often do you run out of medicine?	88 (93.6%)	6 (6.4%)	92 (96.8%)	3 (3.2%)
5. How often do you skip a dose of your medicine before you go to the doctor?	90 (95.7%)	4 (4.3%)	94 (98.9%)	1 (1.1%)
6. How often do you miss taking your medicine when you feel better?	93 (98.9%)	1 (1.1%)	95 (100%)	0
7. How often do you miss taking your medicine when you feel worse?	92 (97.9%)	2 (2.1%)	94 (98.9%)	1 (1.1%)
8. How often do you miss taking your medicine when you are careless?	60 (63.8%)	34 (36.2%)	90 (94.7%)	5 (5.3%)
9. How often do you change the dose of your medicines to suit your needs?	91 (96.8%)	3 (3.2%)	92 (96.8%)	3 (3.2%)
10. How often do you forget to take your medicine when you are supposed to take it more than once a day?	66 (70.2%)	28 (29.8%)	95 (100%)	0
11. How often do you put off refilling your medicines because they cost too much money?	94 (100%)	0	95 (100%)	0
12. How often do you plan ahead and refill your medicines before they run out?*	74 (78.7%)	20 (21.3%)	86 (90.5%)	9 (9.5%)

For the sake of clarity, patients who responded “never” (score 1) were isolated from patients who reported to have non-complete adherence sometimes (score 2), often (score 3), or always (score 4)

^*^For question 12 was used the reverse-code

Considering the 94 patients who reported a low adherence at week-0, the main reason for low adherence was attributed to difficult ruxolitinib supply process (46 patients, 48.9%), as demonstrated by high scores in Q3 and Q12. However, many patients (44 patients, 46.8%) reported mainly an unintentional non-take. Accordingly, intentional low adherence was rare and reported only by 4 (4.3%) patients.

Notably, among the 42 patients who responded to forget to take ruxolitinib sometimes, often, or always (Q1), 24 (57%) also reported that the reason behind such forgetfulness was the twice-daily administration of the drug (Q10).

Globally, the percentage of patients declaring a low adherence was comparable in PV (54.2%) and MF (48.2%) (*p* = 0.48). However, considering only the 4 questions related to unintentional low adherence, this was more frequent in the PV cohort, where 17 out of 26 PV patients (65.4%) declared an unintentional low adherence (vs 27 out of 68 MF patients, 39.7%, *p* = 0.03).

**Fig. 1:**
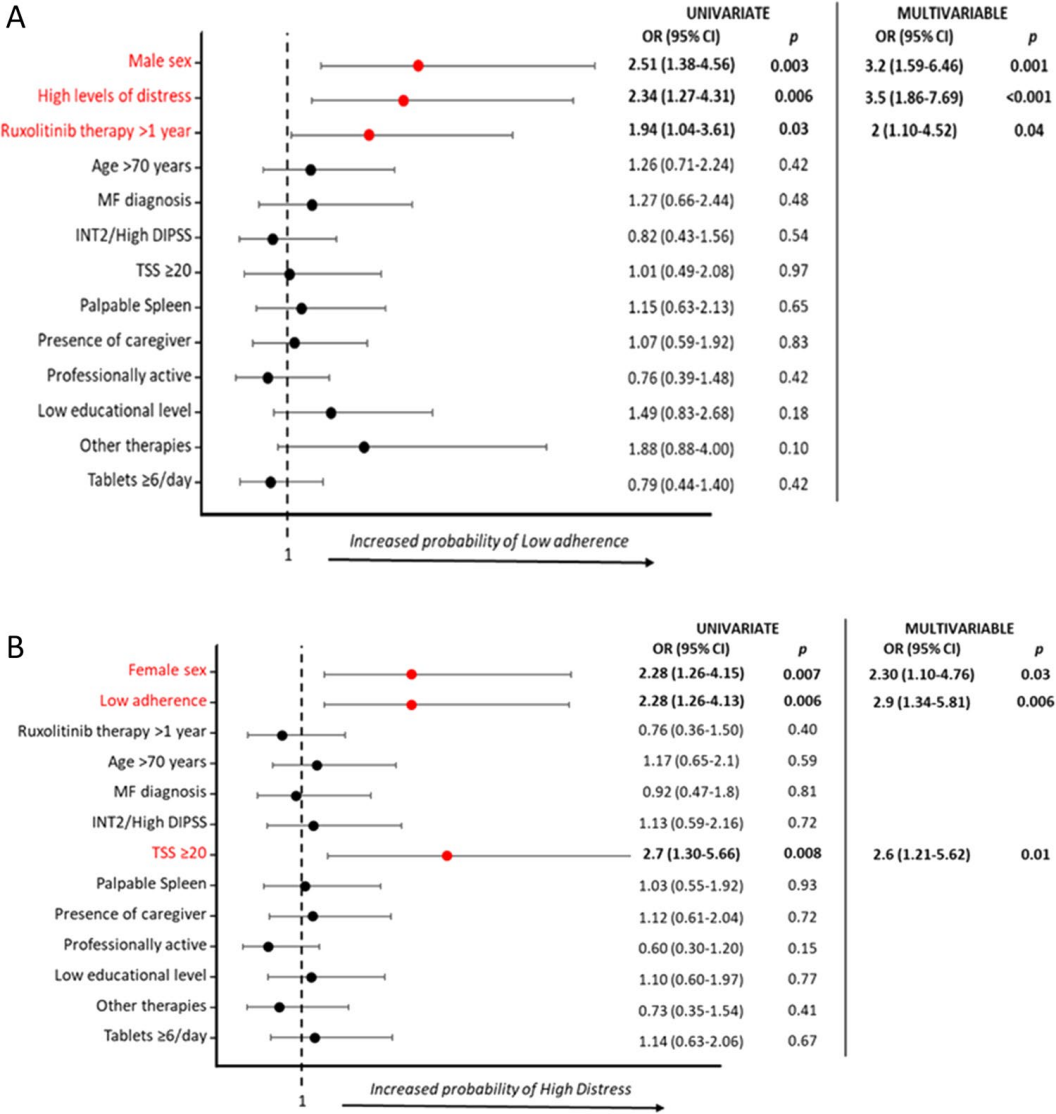
Week-0 characteristics associated with low adherence (**A**) and high distress (**B**)

In UVA, low adherence was associated to male sex (*p* = 0.003), high levels of distress (*p* = 0.006), duration of ruxolitinib therapy > 1 year (1.94, *p* = 0.03). In MVA, all variables maintained their significance (male sex, *p* = 0.001; high distress, *p* < 0.001; duration of ruxolitinib therapy > 1 year, *p* = 0.04) (Fig. 1A).

Considering only MF patients, a low adherence was associated to male sex (OR:3.6, 95%CI:1.29–5.12, *p* = 0.001) and high distress (OR:3.4, 95%CI:1.27–4.31, *p* = 0.002).

In PV patients, low adherence was associated to low educational level (OR:3.7, 95%CI:1.05–13.24, *p* = 0.04).

### Psychological distress at week-0

At week-0, the mean DT score was 3.18 (SD, 2.87). Considering the cut-off value of DT ≥ 4, 76 patients (40.2%) had a high distress. The percentage of patients with high distress was comparable in PV (41.7%) and MF (39.7%) (*p* = 0.81).

In UVA, high distress was associated to female sex (*p* = 0.007), TSS ≥ 20 (*p* = 0.008) and low adherence (*p* = 0.007). In MVA, female sex (*p* = 0.03), TSS ≥ 20 (*p* = 0.01), low adherence (*p* = 0.006), were confirmed as risk factors for high distress (Fig. 1B).

Overall, 4.5% of the patients reported only emotional problems, 28.5% only physical and 55.9% both. Patients with high distress differed from patients with low distress mainly in emotional and physical problems (Supplemental Table 2 and Supplemental Fig. 2).

### Adherence and distress over time

**Fig. 2:**
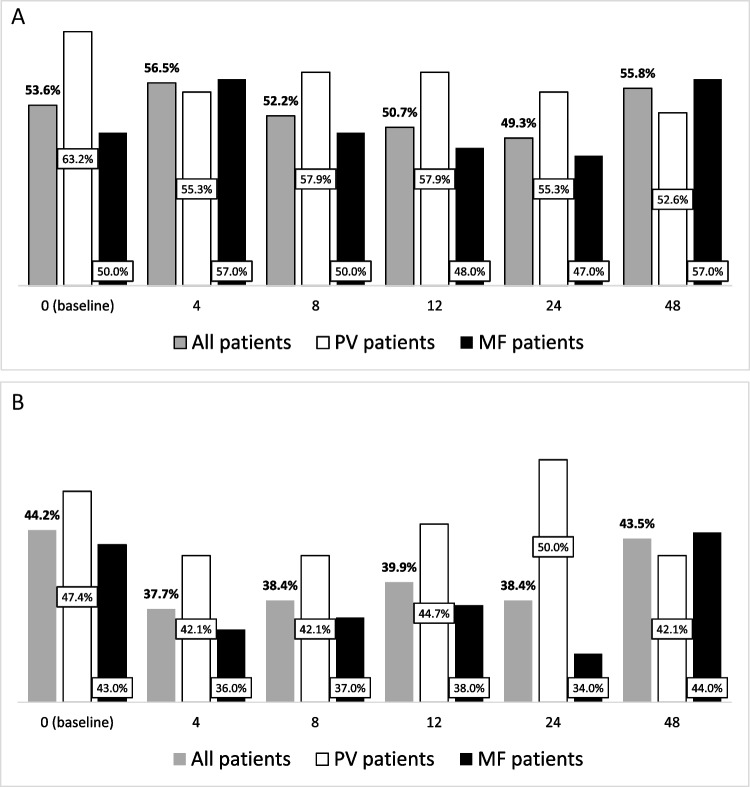
Percentages of 138 “full completers” that declared to have a low adherence (**A**) and a high distress (**B**) overall and according to disease type

Adherence and distress over time were evaluated in the 138 full-completers. The percentages of patients with low adherence (Fig. 2A) and high distress (Fig. 2B) mildly fluctuated during the observation time, ranging between 49.3% to 56.5% and between 37.7% to 44.2%, respectively.

Looking separately at the 3 categories of questions (intentional, unintentional, refill process) among patients who reported low adherence at each timepoint, the percentage of patients who reported unintentional non-take decreased from 47.9% to 26.0% over time. No consistent changes were observed in the other 2 categories of reasons for non-adherence (Supplemental Fig. 3 and Supplemental Table 3).

A total of 51 (36.9%) patients always reported a low adherence through the study. Stable low adherence was more frequent in PV (52.6% vs 31% in MF, *p* = 0.02). In UVA, stable low adherence was associated to male sex (OR:3.64, 95%CI:1.69–7.90, *p* = 0.001), PV diagnosis (OR:2.47, 95%CI:1.15–5.3, *p* = 0.02), high distress (OR:2.0, 95%CI:1.0–4.0, *p* = 0.05). In MVA, male sex (OR:4.6, 95%CI:2.0–10.5, *p* < 0.001) and high distress (OR:3.2, 95%CI:1.44–7.04, p = 0.004) maintained statistical significance. Considering MF and PV patients separately, stable low adherence was associated only to male sex in MF (OR:3.5, 95%CI:1.39–9.0, *p* = 0.008) and to low educational level in PV (OR:6.3, 95%CI:1.63–24.5, *p* = 0.008).

A total of 31 (22.5%) patients always reported a high distress, comparably in MF and PV (*p* = 0.83). In MF patients, TSS ≥ 20 (OR:3.34, 95%CI:1.35–8.30, *p* = 0.009) was associated with a stable high distress.

### Correlation between adherence, distress, outcome, and spleen response in MF patients

Among the 18 patients who discontinued ruxolitinib (n = 8) or died (n = 10) during the 48-week observation time, 17 had a diagnosis of MF. Week-0 low adherence (*p* = 0.14) and high distress (*p* = 0.66) were not associated with subsequent ruxolitinib discontinuation or death. Patients reporting low adherence and high distress at both week-0 and 4 had a slightly higher probability of ruxolitinib discontinuation or death in the following weeks with respect to other patients (HR:1.59, 95% CI:0.44–5.81; *p* = 0.48).

Overall, 61 MF patients always declared a high adherence through week-0 to week-24. At week-0, 26 patients (42.6%) had a spleen response. At week 24, 36 MF patients had maintained or achieved the spleen response, while 25 had lost or failed to obtain the spleen response (*p* = 0.02). Analogously, among the 69 MF patients always declaring a low distress, the probability of maintaining or achieving spleen response was higher (*p* = 0.04) (Table 3).

**Table 3:** Changes in the rates of spleen response in patients with stable high adherence and low distress

		Status	Worsened response	Improved response	Status	P*
		**Week 0 (n. 61)**	**Week 24**			
Spleen response in patients with stable HIGH-ADH	no	35 (57.4%)	n.a	13	25 (41.0%)	***0.02***
	yes	26 (42.6%)	3	n.a	36 (59.0%)	
		**Week 0 (n. 69)**	**Week 24**			
Spleen response in patients with stable LOW-DT	no	36 (52.2%)	n.a	10	28 (40.6%)	***0.04***
	yes	33 (47.8%)	2	n.a	41 (59.4%)	

^*^P-value of McNemar test assessing the variations of spleen response rates between week 0 and week 24

## Discussion

In this study, almost half of the patients reported low adherence to ruxolitinib. This percentage slightly fluctuated during the 48-week observation period and, of the 138 full-completers, 36.9% consistently reported low adherence. This finding is surprising in its magnitude, as the immediate and dose-dependent beneficial effect of ruxolitinib has always suggested a priori near-optimal adherence in most patients. Indeed, ruxolitinib discontinuation may evoke a clinically significant discontinuation syndrome [13]. However, similar findings of low adherence had already been reported in the prospective “ROMEI” study [17].

This study also identified some risk factors for low adherence, including male gender, prolonged (> 1 year) therapy with ruxolitinib, and high levels of psychological distress. Chronic therapies are often associated with reduced compliance and therefore require closer patient monitoring [30]. Notably, 40.2% of patients had a high level of distress at week-0, consistently reported by 22.5% of patients at all timepoints. Female gender and high symptom burden (TSS ≥ 20) at week-0 were found to be the main factors associated with high distress.

These data are consistent with the low quality of life that characterizes all MPN patients, regardless of disease type [31–34]. Accordingly, at week-0 we observed no difference distress and TSS values between the two diseases, highlighting how PV patients started on ruxolitinib can have debilitating symptoms as in MF. The reasons for high distress were mainly related to physical problems, most of which were a direct consequence of the hematological cancer, especially in MF.

High distress and low adherence were found to be correlated. The association between distress and reduced patient self-care including lower medication adherence was observed in other chronic diseases but was never demonstrated in MPNs. This finding may support the importance of integrating psychological support into the management of MPN patients [35].

The main reason for low adherence was the difficulty in obtaining ruxolitinib. While high-cost drugs are dispensed free of charge in Italy, patients have to collect ruxolitinib from the hospital pharmacy every 28 days after a personalized electronic request from the treating hematologist. This system, while ensuring a tightly controlled drug supply, may be difficult to manage for some patients. In PV, low adherence was also more frequent in patients with low levels of education [36]. These findings suggest that providing appropriate support and information to patients according to their health literacy and socioeconomic status may be crucial to improve adherence and reduce distress [37, 38].

Notably, low adherence was unintentional in most cases, and among patients who reported missing doses, the majority attributed this oversight to the twice-daily administration of ruxolitinib. Overall, this is aligned with other reports showing that multiple daily administrations may reduce patient compliance [39]. However, the percentage of patients reporting unintentional low adherence tended to decrease over time. Random variation in these percentages cannot be ruled out. However, both the increased focus of the hematologist on adherence and the serial administration of the questionnaires over time may have had a beneficial impact on patient compliance, reducing unintentional low adherence.

We observed a significant association between stable levels of low adherence/high distress and reduced spleen responses in MF. The promotion of adherence to ruxolitinib and the amelioration of the psychological conditions of MF patients may therefore become a crucial clinical endpoint, since they correlate with better responses, which ultimately lead to more favorable outcome [15]. Accordingly, poor adherence has been associated with increased treatment failure in many diseases, with serious social and economic consequences [16].

We acknowledge the limitations of this study, mainly the self-reported nature of the questionnaires used for the first time in a cohort of MPN patients, the relatively small number of patients included and the different timing of the first ARMS/DTPL assessment. However, these patients were all prospectively followed in dedicated hematology centers and homogeneously treated with ruxolitinib, and the drop-out rate was relatively low for a long-term observational study including patients with chronic malignancies and high disease burden.

To the best of our knowledge, this is the first prospective study evaluating adherence to ruxolitinib and psychological distress over a 48-week period, reported by patients using the distress thermometer alongside the ARMS-scale [18, 21].

We show that low adherence to ruxolitinib represents an unmet clinical need that requires a multifaceted approach based on patient characteristics including gender, health literacy, symptom burden, disease type and treatment duration. Correct assessment of adherence may be relevant to clinical practice, as it may differentiate truly refractory MF patients from those in need of therapy optimization. Strategies to address system and organizational barriers and to improve patient awareness and cooperation are warranted.

### Electronic supplementary material

Below is the link to the electronic supplementary material.

Supplementary file1 (DOCX 374 KB)

## Data Availability

The data that support the findings of this study are available from the corresponding author, F.P., upon reasonable request.

## Appendix

**Table:**

Center	Contributors
IRCCS Azienda Ospedaliero-Universitaria di Bologna, Bologna, Italy / Dipartimento di Medicina Specialistica, Diagnostica e Sperimentale, Università di Bologna, Bologna, Italy	Francesca Palandri Giuseppe Auteri Camilla Mazzoni Marta Venturi Filippo Branzanti Nicola Vianelli Michele Cavo
Department of Scienze Mediche, Chirurgiche e Tecnologie Avanzate “G.F. Ingrassia”, University of Catania, Italy Postgraduate School of Hematology, University of Catania	Giuseppe A. Palumbo Andrea Duminuco
Hematology Division, San Gerardo Hospital, ASST Monza, Italy	Elena M. Elli Alessia Ripamonti
Hematology, S. Eugenio Hospital, Tor Vergata University, ASL Roma2, Rome, Italy	Elisabetta Abruzzese Malgorzata M. Trawinska Vanessa Velotta
Hematology Division, Foundation IRCCS Ca' Granda Ospedale Maggiore Policlinico, Milan	Alessandra Iurlo Daniele Cattaneo
Section of Hematology, University of Verona, Verona, Italy	Massimiliano Bonifacio Mauro Krampera Luigi Scaffidi Andrea Bernardelli
Ematologia, Ospedale Businco, Università degli studi di Cagliari, Cagliari, Italy	Giovanni Caocci Maria Pina Simula Olga Mulas Alessandro Costa
U.O.C. di Ematologia, Department of Hemato-Oncology, Azienda Ospedaliera Annunziata	Francesco Mendicino Enrica A. Martino
Hematology Unit, Ospedale Belcolle, Viterbo, Italy	Roberto Latagliata
