# Supplementary material for: Ruxolitinib Adherence in Myelofibrosis and Polycythemia Vera: the “RAMP” Italian multicenter prospective study
Source: Ann Hematol. 2024 Mar 13;103(6):1931–40. doi: 10.1007/s00277-024-05704-0 (PMC11090921; doi:10.1007/s00277-024-05704-0)
Supplement: Supplementary file 1 — Supplementary file1 (DOCX 374 KB) [file 277_2024_5704_MOESM1_ESM.docx]

## Supplemental Figure 1: Patients’ disposition


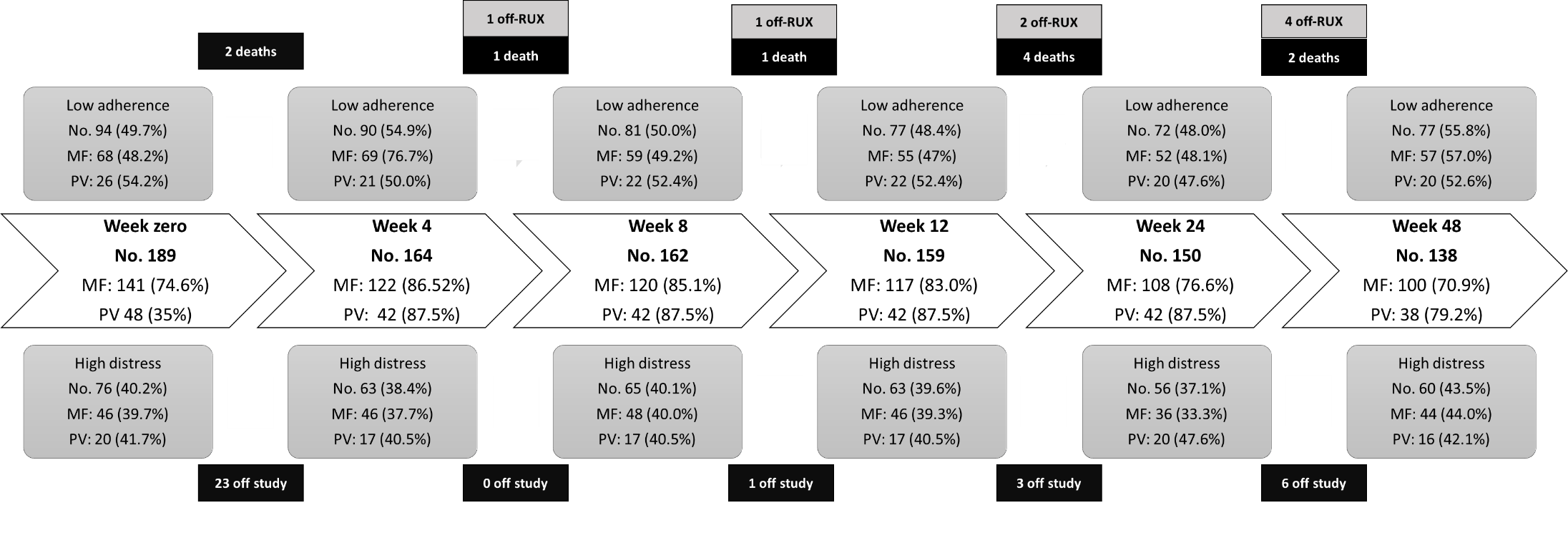


| **Characteristics at week 0** | **No. of pts = 189** |
| --- | --- |
| **Presence of caregiver**, n (%) | 85 (45.0%) |
| **Education**, n (%)  None  Primary  Middle school  High school  University | 2 (1.0%)  31 (16.4%)  55 (29.1%)  74 (39.2%)  27 (14.3%) |
| **Employment**, n (%)  Retired  Employed  Domestic work  Unemployed | 105 (55.5%)  50 (26.6%)  30 (15.8%)  4 (2.1%) |
| **Visit frequency in the last 12 months**  One or more visit every 3 months  One visit every 3-6 months  One visit > 6 months | 52 (27.6%)  132 (69.8%)  5 (2.6%) |
| **Other therapies for other diseases**, n (%)  Total number of daily tablets except ruxolitinib, median (range)  Patients taking > 6 tablets a day except ruxolitinib, no (%) | 152 (80.4%)  6 (0-20)  94 (49.7%) |
| **Patients who are followed by a stable team of hematologists**, no. (%) | 189 (100%) |
| **Patients who are satisfied by the relationship with the hematologists**, no. (%) | 188 (99.5%) |
| **Patients who feel they are taking an excessive number of tablets/daily**, no. (%) | 19 (10%) |
| **Patients who feel that ruxolitinib intake impairs their work/social activities**, no. (%) | 7 (3.7%) |
| **Patients who feel that correct ruxolitinib intake is important for their health**, no (%) | 186 (98.4%) |

## Supplemental Table 1: Basic patients’ information

Supplemental Table 2: Problem list at week-0, according to degree of distress and disease type
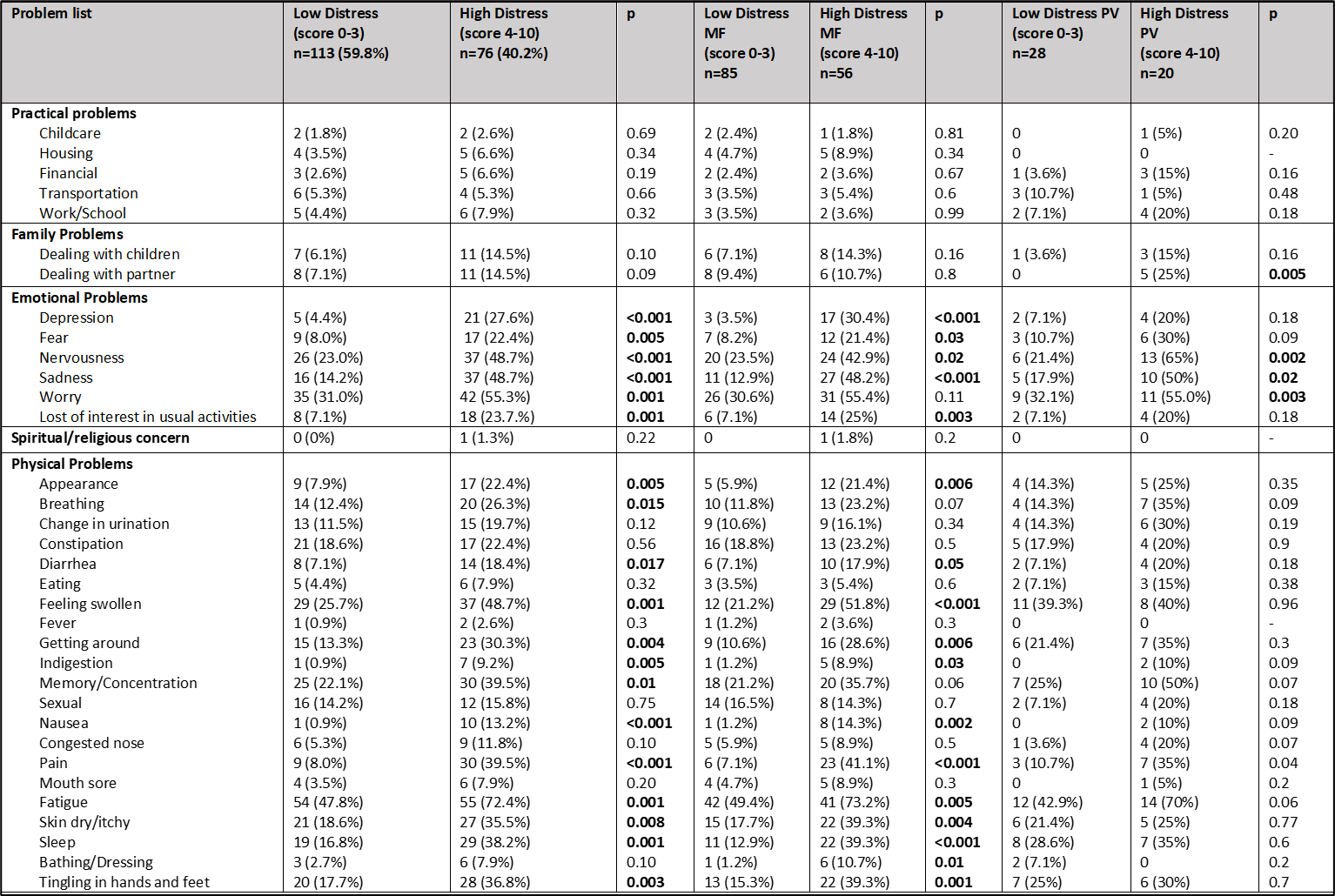


Supplemental Figure 2: Main problems with differential expression in patients with high and low distress

## Supplemental Figure 3: Reasons for Low adherence

Supplemental Table 3: mean ARMS total scores and mean distress thermometer total scores

| **ARMS** | |
| --- | --- |
| **Timepoint** | **Mean ± SD [range]** |
| Week 0 | 14.35 ± 2.02 [12-21] |
| Week 24 | 14.12 ± 1.95 [12-22] |
| Week 48 | 14.39 ± 1.88 [12-21] |
| **Distress Thermometer** | |
| **Timepoint** | **Mean ± SD [range]** |
| Week 0 | 3.18 ± 2.87 [0-10] |
| Week 24 | 2.74 ± 2.52 [0-10] |
| Week 48 | 3.18 ± 2.72 [0-10] |
